# Dengue virus infection in a French traveller to the hilly region of Nepal in 2015: a case report

**DOI:** 10.1186/s13256-016-0847-1

**Published:** 2016-03-21

**Authors:** Birendra Prasad Gupta, Anurag Adhikari, Ramanuj Rauniyar, Roshan Kurmi, Bishnu Prasad Upadhya, Bimlesh Kumar Jha, Basudev Pandey, Krishna Das Manandhar

**Affiliations:** 1 Virology Unit, Central Department of Biotechnology, Tribhuvan University, Kritipur, Kathmandu, Nepal; 2 Asian Institute of Technology and Management, Purbanchal University, Lalitpur, Nepal; 3 Everest School of Applied Science, Kathmandu, Nepal; 4 Bhawani Hospital and Research Center, Birgunj, Nepal; 5 National Public Health Laboratory, Teku, Kathmandu, Nepal; 6 Leprosy Division, Ministry of Health, Government of Nepal, Kathmandu, Nepal

**Keywords:** Dengue virus, Hilly region, Nepal, Travel

## Abstract

**Background:**

Dengue viral infections are known to pose a significant risk during travel to tropical regions, but it is surprising to find dengue transmission in the hilly region of Nepal, which is over 1800mtr above sea level.

**Case presentation:**

A 43-year-old Caucasian female traveler from France presented with fever and abdominal pain following a diarrheal illness while visiting the central hilly region of Nepal. Over the course of 9 days, she developed fever, body aches, and joint pain, with hemorrhagic manifestation. She was hospitalized in India and treated with supportive care, with daily monitoring of her platelets. An assessment by enzyme-linked immunosorbent assay showed that she was positive for dengue non-structural protein 1. Upon her return to France, dengue virus was confirmed by reverse transcriptase-polymerase chain reaction.

**Conclusion:**

The district where this dengue case was reported is in the hilly region of Nepal, neighboring the capital city Kathmandu. To the best of our knowledge, there has previously been no dengue cases reported from the district. This study is important because it aims to establish a potential region of dengue virus circulation not only in the tropics, but also in the subtropics as well, which in Nepal may exceed elevations of 1800mtr. This recent case report has raised alarm among concerned health personnel, researchers, and organizations that this infectious disease is now on the way to becoming established in a temperate climate.

## Background

Dengue is a mosquito-borne flaviviral infection that is endemic to the tropics and subtropics, affecting up to 100 million people per year [1]. The presentation of dengue virus (DENV) infection can be asymptomatic or manifest as a febrile illness; dengue fever. However, subsets of patients may develop dengue hemorrhagic fever, with complications such as gastrointestinal bleeding and ecchymoses, which may result in the potentially lethal form of the disease; dengue shock syndrome [2–4]. Around the world there are four distinct dengue virus serotypes (DENV 1–4) [5]. Interestingly, infection with one serotype confers serotype-specific immunity to the host; however, if subsequent infection occurs with a different serotype, it can predispose the host to a grave clinical outcome [6–8].

Nepal shares an open border with India, an endemic region for DENV, thus is susceptible to an unprecedented flow of virus through infected patients crossing the border. Regulatory checkpoints and quarantines are not currently in place along the Nepali border. In 2013, a Nepali team isolated and characterized DENV circulating in a part of the eastern Terai that had previously seen cases of dengue in 2006 [9]. Comparing the data, Singh *et al.* [10] reported that, in fact, there were two different strains of DENV circulating in Nepal, raising the specter of an alarming dengue situation in Nepal.

We report a case of DENV infection in a Caucasian woman while she was traveling through the hilly region of Nepal.

## Case presentation

A 43-year-old Caucasian female arrived in Kathmandu from Paris, and the same day traveled to Kavre District, in the inner hilly region (elevation: 1800mtr) of Nepal. Nine days after arriving in the hilly region, she developed fever, body aches and joint pain. She also experienced loose stools and vomiting along with hemorrhagic manifestations such as facial flushing, petechiae and edema. She was subsequently admitted to a hospital in Goa, India, owing to health insurance requirements. Upon examination, her blood pressure and other vital signs were normal, without abdominal distension or bleeding. Ultrasonography of her abdomen and pelvis showed borderline splenomegaly (12cm). An enzyme-linked immunosorbent assay was positive for dengue non-structural protein 1, although tests for immunoglobulins G and M were negative. She was given supportive care, including intravenous fluids. Daily monitoring of her platelet count revealed values as low as 37,000/mm^3^ (Table 1). She was discharged from the hospital in Goa after 5 days, and returned to Paris, where she was hospitalized briefly and discharged. Dengue virus was confirmed by reverse transcriptase-polymerase chain reaction in Paris.

**Table 1:** Laboratory parameters of dengue case

Test	Result
Hemoglobin	13.6 g/dL
Packed cell volume	39.1 L/L
Platelets	37,000/mm^3^
Total bilirubin	0.9 mg/dL
Direct bilirubin	0.4 mg/dL
Serum glutamic oxaloacetic transaminase	93 IU/L
Serum glutamic-pyruvic transaminase	54 IU/L
Alkaline phosphatase	53 IU/L
Total proteins	7.7 g/dL
Albumin	4 g/dL
Globulin	3.7 g/dL
Albumin to globulin Ratio	1.08
Gamma-glutamyl transferase	45 IU/L
Dengue immunoglobulin G and M	Negative
Dengue non-structural protein 1 antigen	Positive

## Discussion

The first case of DENV2 infection in Nepal was reported in 2004 in a Japanese traveler [11]. Subsequently, confirmed cases were reported from nine districts in the lowland Terai region of Nepal in 2006, and all four DENV serotypes were found to be co-circulating that year [9]. Over the years, the Terai belt, located south of the Himalayan foothills, has become an established dengue region. However, only limited data have been generated about DENV infection and disease in Nepal. Two major outbreaks have occurred in Nepal: one in 2010, dominated by DENV1, and the other in 2013, dominated by DENV2 [10, 12]. Dengue was reported in the highlands for the first time during the 2010 outbreak [12]. The 2013 outbreak also affected the highlands, including Kathmandu, though it was predominantly confined to Chitwan, Rupandehi, Parsa, and Dhanusa [10]. The shift of the viral disease from the subtropical Terai belt to the temperate hill region of Nepal is relevant to medical science because the disease could easily spread all over the world in the future, irrespective of climate [13]. The village to which the  patient had traveled is at an elevation of 1800mtr. It is becoming apparent that DENV is adapting to temperate climates as well. This may be because of temperature change due to global warming, the resistance of mosquitoes to drugs, or other factors, all of which may have contributed to the increase in dengue fever cases around the world in recent years.

Reports of dengue among travelers worldwide have been increasing, but high-quality data on incidence are lacking. The increase in international travel to dengue-endemic regions, with the associated risk of travel-acquired dengue, poses a serious concern [14]. The shift in temperature around the world has enabled DENV to thrive in colder regions where it was previously not reported. To look for the causes, the mosquito vector *Aedes* spp. and mutations in the virus should be the foci of future research. *A. aegypti* was first reported in Kathmandu in 2010 and the vectors may have now established populations up in the mountains [12]. Serotype and genotype shifts have been reported based on sequencing of specific viral regions [15]. In-depth molecular study of the virus found in the hill region of Nepal might give insights into the climatic shift of dengue disease. Therefore, the authors strongly recommend active surveillance and identification of mosquitoes along with whole genome sequencing of the virus for greater awareness as well as knowledge of dengue fever and dengue hemorrhagic fever transmission.

## Conclusion

This is the second report of DENV infection in a foreigner travelling in Nepal, after the case in a Japanese traveler reported in 2004. Although initially it was thought that only the Terai belt of Nepal was endemic for dengue, a number of dengue cases are also being reported from the hill region. The Government of Nepal should take the initiative to eliminate adult mosquitoes, as well as larvae and eggs, to prevent further transmission of arboviruses in the hill region of Nepal, which is one of the most attractive touristic sites in Nepal.

## Consent

Written informed consent was obtained from the patient for publication of this case report and any accompanying images. A copy of the written consent is available for review by the Editor-in-Chief of this journal.

## References

[1] Kosasih H, Alisjahbana B, Nurhayati, de Mast Q, Rudiman IF, Widjaja S, et al. The epidemiology, virology and clinical findings of dengue virus infections in a cohort of Indonesian adults in Western Java. PLoS Negl Trop Dis. 2016;10(2):e0004390. 10.1371/journal.pntd.0004390 PMC4752237 26872216

[2] Zhang H, Zhou YP, Peng HJ, Zhang XH, Zhou FY, Liu ZH, et al. Predictive symptoms and signs of severe dengue disease for patients with dengue fever: a meta-analysis. Biomed Res Int. 2014;2014:359308. 10.1155/2014/359308 PMC4100454 25097856

[3] John DV, Lin YS, Perng GC. Biomarkers of severe dengue disease - a review. J Biomed Sci. 2015;22:83. 10.1186/s12929-015-0191-6 PMC4604634 26462910

[4] Sharma K, Yadav A. Association of mean platelet volume with severity, serology & treatment outcome in dengue fever: prognostic utility. J Clin Diagn Res. 2015;9(11):EC01–03. 10.7860/JCDR/2015/14285.6710 PMC4668412 26673082

[5] Henchal EA, Putnak JR. The dengue viruses. Clin Microbiol Rev. 1990;3(4):376–96. 10.1128/cmr.3.4.376 PMC358169 2224837

[6] Guzman MG. Global voices of science. Deciphering dengue: the Cuban experience. Science. 2005;309(5740):1495–7. 10.1126/science.1115177 16141052

[7] Halstead SB. Neutralization and antibody-dependent enhancement of dengue viruses. Adv Virus Res. 2003;60:421–67. 10.1016/s0065-3527(03)60011-4 14689700

[8] Endy TP, Nisalak A, Chunsuttitwat S, Vaughn DW, Green S, Ennis FA, et al. Relationship of preexisting dengue virus (DV) neutralizing antibody levels to viremia and severity of disease in a prospective cohort study of DV infection in Thailand. J Infect Dis. 2004;189(6):990–1000. 10.1086/382280 14999601

[9] Malla S, Thakur GD, Shrestha SK, Banjeree MK, Thapa LB, Gongal G, et al. Identification of all dengue serotypes in Nepal. Emerg Infect Dis. 2008;14(10):1669–70. 10.3201/eid1410.080432 PMC2609890 18826846

[10] Singh S, Gupta BP, Manakkadan A, Das Manandhar K, Sreekumar E. Phylogenetic study reveals co-circulation of Asian II and Cosmopolitan genotypes of dengue virus serotype 2 in Nepal during 2013. Infect Genet Evol. 2015;34:402–9. 10.1016/j.meegid.2015.07.006 26160542

[11] Takasaki T, Kotaki A, Nishimura K, Sato Y, Tokuda A, Lim CK, et al. Dengue virus type 2 isolated from an imported dengue patient in Japan: first isolation of dengue virus from Nepal. J Travel Med. 2008;15(1):46–9. 10.1111/j.1708-8305.2007.00165.x 18217869

[12] Pandey BD, Nabeshima T, Pandey K, Rajendra SP, Shah Y, Adhikari BR, et al. First isolation of dengue virus from the 2010 epidemic in Nepal. Trop Med Health. 2013;41(3):103–11. 10.2149/tmh.2012-17 PMC3801155 24155651

[13] Dhimal M, Gautam I, Kress A, Muller R, Kuch U. Spatio-temporal distribution of dengue and lymphatic filariasis vectors along an altitudinal transect in Central Nepal. PLoS Negl Trop Dis. 2014;8(7), e3035. 10.1371/journal.pntd.0003035 PMC4117448 25078276

[14] Dhimal M, Gautam I, Joshi HD, O’Hara RB, Ahrens B, Kuch U. Risk factors for the presence of chikungunya and dengue vectors (*Aedes aegypti* and *Aedes albopictus*), their altitudinal distribution and climatic determinants of their abundance in central Nepal. PLoS Negl Trop Dis. 2015;9(3), e0003545. 10.1371/journal.pntd.0003545 PMC4361564 25774518

[15] Gupta BP, Singh S, Kurmi R, Malla R, Sreekumar E, Manandhar KD. Re-emergence of dengue virus serotype 2 strains in the 2013 outbreak in Nepal. Indian J Med Res. 2015;142 Suppl: S1–6. 10.4103/0971-5916.176564 PMC4795338 26905233

